# Early embryonic development and spatiotemporal localization of mammalian primordial germ cell-associated proteins in the basal rodent *Lagostomus maximus*

**DOI:** 10.1038/s41598-017-00723-6

**Published:** 2017-04-04

**Authors:** Noelia P. Leopardo, Alfredo D. Vitullo

**Affiliations:** 1 Departamento de Ciencias Biomédicas y Biotecnológicas, Centro de Estudios Biomédicos, Biotecnológicos, Ambientales y Diagnóstico -CEBBAD-, Universidad Maimónides, Hidalgo 775, C1405BCK, Buenos Aires, Argentina; 2 Consejo Nacional de Investigaciones Científicas y Técnicas, CONICET, Buenos Aires, Argentina

## Abstract

The gene network controlling primordial germ cell (PGC) specification in eutherian mammals has been exhaustively investigated in mice. The egg-cylinder morphology of the mouse embryo is the key event enabling inductive signals from the extra-embryonic ectoderm (ExE) to specify epiblast cells as PGCs early on. We investigated the embryonic development and the spatiotemporal localization of PGC-associated proteins in the basal Hystricognathi rodent *Lagostomus maximus*. *L. maximus* develops through a flat-disc epiblast far apart from the ExE. In the primitive streak stage, OCT4-positive cells are detected in the posterior pole of the embryo disc in the mesoderm of the proximal epiblast. In the neural plate stage, a reduced 8 to 12 OCT4-positive cell population transiently expresses *FRAGILIS*, *STELLA* and *SOX17* in the posterior streak. Soon after translocation to the hindgut, pluripotent OCT4 cells start expressing *VASA*, and then, *STELLA* and *FRAGILIS* are turned on during migration toward the genital ridge. *L. maximus* shows a spatiotemporal pattern of PGC-associated markers divergent from the early PGC restriction model seen in mice. This pattern conforms to alternative models that are based on a pluripotent population in the embryonic axis, where PGCs are specified later during development.

## Introduction

The molecular machinery of primordial germ cell (PGC) specification has been studied in laboratory mice and it has erected as the paradigm for germline development in eutherian mammals^1^. The appearance of PGCs in mice depends on the sequential and overlapping expression of a group of molecules responsible for the specification and differentiation of the germ line, making cells competent to receive specific signals and preventing them from retaining the characteristics of somatic cells^2–6^. These cells are thought to originate from the most proximal epiblast by induction from the extraembryonic ectoderm (ExE) and the visceral endoderm (VEn). Both extraembryonic tissues surround the epiblast cells of the post-implantation egg cylinder embryo from approximately E5.0 to E6.0. The ExE and VEn release the bone morphogenetic proteins (Bmp) 4, 8b and 2 to the surrounding tissue to instruct a small number of pluripotent proximal epiblast cells to become competent to be PGCs, suppressing the somatic program that is adopted by neighboring cells. The high levels of Bmp activate the expression of *Fragilis* and competent cells acquire the ability to form PGCs. Within these *Fragilis*-positive cells, approximately 6 cells begin to express *Blimp1* and *Prdm14* at around E6.25^7, 8^. *Blimp1* has a critical role in the foundation of the mouse germ cell lineage since its disruption causes an early block in the process of PGC formation^5, 8^. *Blimp1*-expressing cells increase in number from 6 to 16 at approximately E6.5. By E6.75 to E7, 20–28 cells move posteriorly and develop alkaline phosphatase activity and *Stella* expression. During early gastrulation (E7.25), the PGCs form a cluster of approximately 40 cells at the base of the incipient allantois in the extraembryonic mesoderm (ExM)^9, 10^. From E7 to E7.75, they regain expression of pluripotency-associated genes, such as *Oct4, Sox2*, *Nanog* and *Stella*, and suppress the expression of genes involved in mesodermal specification. Subsequently and concomitant with an increase in their number, PGCs start to migrate one by one through the developing hindgut endoderm. They then exit the endoderm to reach the mesentery, and at approximately E10.5, they colonize the genital ridges, where they proliferate and differentiate into oogonia or spermatogonia^11^.

Whether the mouse regulatory pathway is the established mechanism of germ line formation in other (or all) mammals remains largely unexplored^1^. There are some key embryological differences between mice and other mammals, especially at the epiblast stage, when PGCs are thought to be specified. In mice, the sequential gene expression of PGC specification depends on the topographical proximity between cells of different embryonic tissues, due to the fact that the epiblast forms a cup-shaped egg cylinder, allowing the most proximal epiblast cells to be in close contact with the ExE. However, the early embryo morphology that most mammals have is a flat disc-like epiblast, the mammotypical early embryo morphology^12^, which clearly departs from the mouse egg cylinder. In the flat disc of non-rodent embryos, such as humans, pigs and rabbits, the epiblast contacts with the VEn, and ExE is absent. Moreover, rodents from taxonomic groups other than murids also display a flat disc embryo. This is the case in guinea-pigs (*Cavia porcellus*) in which the embryo is cup-shaped similar to in mice but the ExE does not contact the epiblast before or after grastrulation^13^. This topographical embryo difference between mice and other mammals, including non-muroid rodents, makes the search of a general mechanism for PGC specification based on the well-established mouse molecular regulatory path difficult.

The mouse pathway complies with the currently accepted model of PGC formation as an early lineage-restricted cluster of cells in the base of the allantois. Nevertheless, no definitive proof demonstrating the continuity of those early-specified PGC and germ cells in gonads has so far been provided, as reviewed by Mikedis and Downs^14^. These authors propose an alternative model in which the alleged PGCs in the base of the allantois are instead a pool of pluripotent progenitor cells in the posterior end of the primitive streak that builds the fetal-placental interface. The pluripotent cell pool condenses into the allantoic core domain (ACD), which extends the body axis posteriorly through the allantoic midline^15^. ACD pluripotent cells express all PGC markers and contribute to both embryonic and extraembryonic tissues^14^. From this pluripotent population, it is suggested that PGC could be segregated later, once evolutionarily conserved genes of germline development, such as *VASA*, *Dazl* and *Nanos*, begin to be expressed^14^. Although this alternative explanation is proposed for the mouse egg cylinder, it may well apply in flat embryos where the ExE is absent or far apart from the epiblast.

The South American plains vizcacha, *Lagostomus maximus*, is a New World Hystricognathi (Caviomorpha) fossorial rodent and a close evolutionary relative of *Cavia porcellus*, inhabiting the southern region of the Neotropics. In this study, we analyzed the post-implantation development in *L. maximus* embryos and showed that they develop through a flat embryonic disc, in which no contact between epiblast and the ExE occurs before or after gastrulation. Moreover, we show that the sequential expression of germ line markers diverges from that in mice before and after gastrulation and during migration toward the developing gonads and colonization of the genital ridges. The spatiotemporal pattern of germ line markers in *L. maximus* conforms to the proposal of a pluripotent cell population within the embryonic axis, from which PGCs may become restricted at later stages during migration^14^.

## Results

### Overview of the implantation development of the plains vizcacha embryo

*L. maximus* embryo has a cup-shaped morphology with a flat disc-like epiblast. The external features of the embryo during the whole gestation period (147 ± 5days) were analyzed and classified (Table S1). Embryo stages classified according to the external morphology and showing the changes that occur in the topographical appearance of the embryo from the pre-somite to the >60 pairs of somite stages, are shown and described in Figure S1.

### Early post-implantation development

#### Implantation site

**Figure 1:**
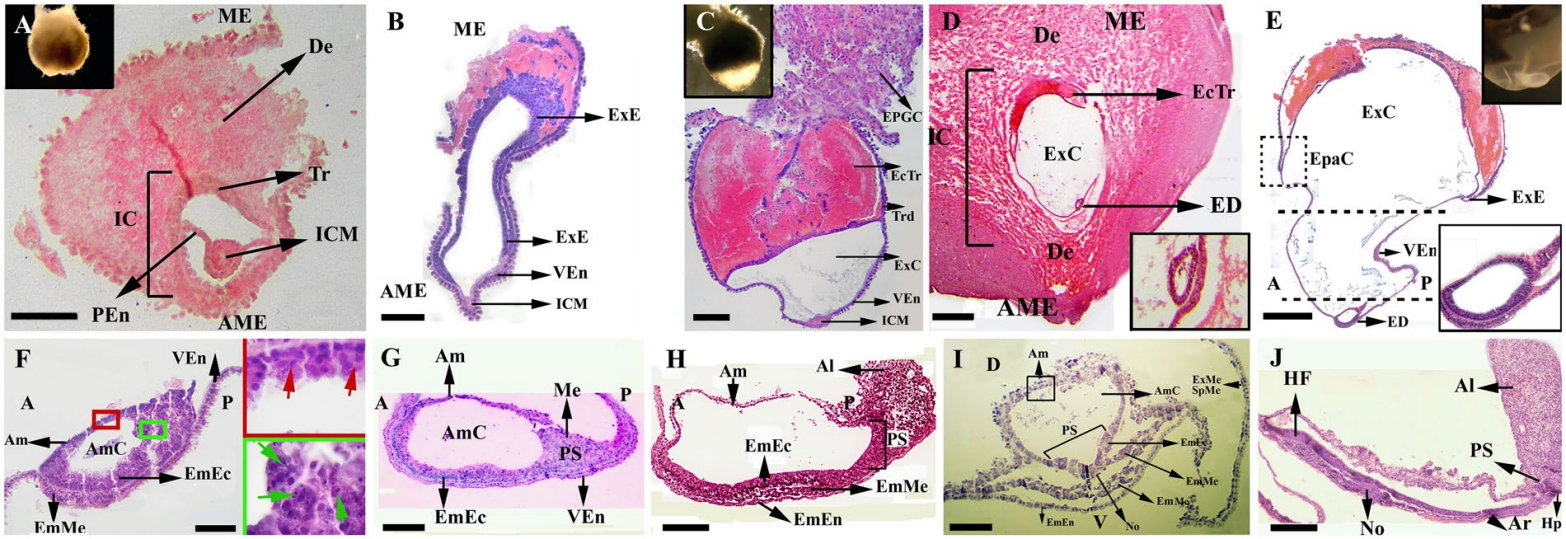
Sagittal sections of the early post-implantation embryo of *Lagostomus maximus*. (**A**) Newly implanted embryo (22–26 days post-fertilization). (**B**) Pre-streak stage, elongated embryo; note that the embryonic and extra-embryonic tissues are far apart. (**C**) Differentiation of the embryo (26–32 days post-fertilization) with the ectoplacental trophoblast invaded by maternal blood; exocoelomic cavity formed. Note the mesometrially situated, ectoplacental giant cells (EPGC). (**D**) General view of the implantation chamber, with the embryo disc at the anti-mesometrial pole (26–32 days post-fertilization). Inset: detail of the embryonic disc. (**E**) Embryonic disc with allantoic bud in the neural plate stage. Note the extraembryonic ectoderm far apart from the cavitated epiblast, separated by the visceral endoderm (dotted lines). Inset: detail of the embryonic disc. (**F**) Embryonic cavitation; upper inset: cells undergoing apoptosis in the amniotic cavity (red arrows); lower inset: cells in apoptosis in the epiblast (green arrows). (**G**) Primitive streak stage: appearance of some mesoderm cells in the posterior region of the epiblast that lines the visceral endoderm. (**H**,**I**) Neural plate stage (32–39 days post-fertilization): appearance of the node. (**J**) Late head-fold stage: foregut invagination, the extension of the allantois. A: anterior; Al: allantois; Am: amnion; AmC: amniotic cavity; AME: antimesometrial; Ar: archenteron; D: dorsal; De: decidua; EcTr: ectoplacental trophoblast; ED: embryonic disc; EmEc: embryonic ectoderm; EmEn: embryonic endoderm; EmMe: embryonic mesoderm; Ep: epiblast; EpaC: epamniotic cavity; EPGC: ectoplacental giant cells; ExC: exocoelomic cavity; ExE: extraembryonic ectoderm; ExMe: extraembryonic mesoderm; Hp: hindgut pocket; IC: implantation chamber; ICM: inner cell mass; ME: mesometrial; No: node; P: posterior; PEn: primitive endoderm; PS: primitive streak; SpMe: splanchnic mesoderm; Tr: trophectoderm; Trd: trophoblast-derived cells; V: ventral; VEn: visceral endoderm. Scale bar: 100 µm.

At 22–26 days of gestation, 6 to 12 implantation sites (IS) distributed in both uterine horns were normally found (Figure S2A). IS were visible externally as spherical swellings, measuring approximately 4 cm in diameter (Table S1 and Figure S2A). The pattern of implantation was interstitial, with the embryo disc implanted in an anti-mesometrial orientation (Figure S2B). The embryo was a double-layered structure composed of an ICM of ectoderm cells (the epiblast) and an outer layer of endoderm cells, the visceral endoderm (VEn). The VEn was in close contact with the ICM, elongating and separating the embryo from the trophoblast. The trophectoderm was in contact with the VEn (Fig. 1A).

#### Differentiation of the embryo

At 26–32 days of gestation, the embryo enlarged considerably and bulged cone-like into the segmentation cavity (Fig. 1B). In this pre-streak stage, evidence of the embryonic axis and the distinction between the embryonic and extraembryonic tissue (ExE) first appeared (Fig. 1B,C). Cone-like structures consisted of unilaminar VEn and supported the ICM. The single epithelium of the VEn extended from beneath the ICM to the ectoplacental trophoblast (Fig. 1B,C). The ExE was far apart from the ICM, separated by the VEn, resulting in a cup-shaped structure (Fig. 1B–E). The exocoelomic cavity formed in the elongated VEn, and the ICM was flattened (Fig. 1B,C). The embryo sac was composed of the ectoplacental trophoblast, which enclosed the epamniotic cavity (Fig. 1E). While the outer lamina bounding this cavity was a simple epithelium, the inner lamina appeared to vary from two to three cells in thickness (Fig. 1B,E). The ectoplacental cavity formed in the ectoplacental trophoblast and was filled with extravasated maternal blood (Fig. 1B–D). No ectoplacental cone (EPC) was identified; the embryo was a bilaminar disc (Fig. 1D) with a rim of mesometrial situated ectoplacental giant cells (EPGC) (Figs 1C,D and S1D,E).

#### Cavitation, gastrulation and formation of the amniotic cavity

In the advanced stage of 32–39 gestation days, the embryo was a cluster of undifferentiated cells, with a cleft in the center forming an incipient amniotic cavity (Fig. 1F). The amniotic cavity formed by cavitation, and the epiblast cells died by apoptosis (Fig. 1F). The cavitated amnioembryonic mass was not in contact with the ExE; the central lumen, or the amniotic cavity, appeared before gastrulation (Fig. 1D), and the embryo was a typical bilaminar disc (Fig. 1D,F). Before the appearance of the allantoic bud, the embryo developed the ectoplacental, amniotic and exocoelomic cavities (Fig. 1D). After cavitation, at the primitive streak stage, there was no significant evidence of differentiation among the cells in the embryonic disc, but mesoderm cells differentiated in the posterior part of the embryo, indicating the beginning of gastrulation (Fig. 1G). At the neural plate stage, a marked swelling resulted in the allantois at one edge of the disc and head folding at the opposite edge. A trilaminar embryonic disc, including the three primary germ layers, ectoderm, mesoderm and endoderm, was established (Fig. 1H). At this time-point, the primitive streak, the notochord and the neural tube were formed. The mesoderm appeared and spread outward between the ectoderm and the endoderm of the embryonic disc. It covered the amniotic ectoderm with somatic mesoderm and extended over the visceral endoderm (yolk sac) with splanchnic mesoderm (ExM), and the ectodermal tissue thickened and flattened into the neural plate (Fig. 1I). At the early head fold pre-somite stage, a hindgut invagination was observed, the allantois extended and the head folded (Fig. 1J and S1F,G).

#### Gene expression during germ cell specification and migration

**Figure 2:**
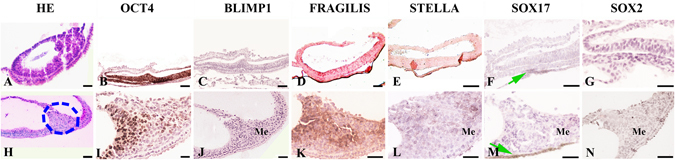
Expression and localization of germ line molecular markers in the embryo disc and primitive streak stage in *Lagostomus maximus*. (**A**) General view of the embryo disc (hematoxylin-eosin staining) and (**H**) primitive streak stage. (**B**) Widespread OCT4 positive expression in the ectoderm of the embryo disc. (**C**) BLIMP1, (**D**) FRAGILIS and (**E**) STELLA were not detected at the embryonic disc stage. (**F**) SOX17 detection in the visceral endoderm of the embryonic disc (green arrow). (**G**) Undetectable SOX2. (**I**) OCT4 positive migratory mesoderm cells during gastrulation (**K**) also expressing FRAGILIS in the primitive streak stage. (**J**) BLIMP1, (**L**) STELLA, (**M**) SOX17 and (**N**) SOX2 were not detectable in mesoderm cells in the primitive streak stage. Note that SOX17-expressing cells were found in the visceral endoderm (green arrow in M). **I**–**N**: detail of the circled area in H. Scale bar: 20 µm (**A**–**D**,**H**,**J**); 40 µm (**E**–**G**,**I**,**K**–**N**).

The expression of *OCT4* was uniformly observed in all epiblast cells (embryonic ectoderm) of the bilaminar embryonic disc in the pre-streak stage (Figs 2B and 6A,D). Except for a few positive cells, the amnion tissue was mostly negative. Interestingly, during this period, BLIMP1, STELLA, FRAGILIS, and SOX2 proteins were undetectable; however, SOX17- immunoreactive cells were found in the VEn (Fig. 2C–G).

As the embryonic development progressed to gastrulation in primitive streak, OCT4-positive ectoderm cells migrated to the posterior pole of the embryo disc to form mesoderm. The number of OCT4-positive cells decreased dramatically to a minimum of 8 to 12 cells at the neural plate stage (Table 1) and was observed to increase again at the late head-fold stage and especially after translocation to the hindgut. *FRAGILIS* expression was observed in the mesoderm cells of the proximal epiblast (Fig. 2K). During this period, the expression of *BLIMP1*, *STELLA*, *SOX2* and *SOX17* was undetectable (Fig. 2J,L–N).

**Table 1:** Germ line marker detection and quantification of OCT4-positive cells throughout embryonic development in *L. maximus*.

GERM LINE MARKER								
Embryo stage	OCT4	VASA	FRAGILIS	STELLA	SOX2	SOX17	BLIMP1	Putative PGC/Germ cell* (means ± S.D.)
Embryo disc	+	−	−	−	−	−	−	ND
Primitive streak	+	−	−	−	−	−	−	ND
Neural Plate	+	−	+	+	−	+	−	8 ± 2^a^
Late Head fold	+	−	−	−	−	+	−	25 ± 5^b^
8–12S	+	+	−	−	−	−	−	98 ± 10^c^
13–20S	+	ND	ND	ND	ND	ND	ND	502 ± 103^d^
35–39S	+	+	+	+	−	−	−	1118 ± 131^e^
45–55S	+	+	+	+	−	−	−	10.532 ± 102^f^
Fetal ovary	+	+	+	+	−	+	−	56. 125 ± 13 .322^g^

*The number of putative PGCs/germ cells was estimated on the basis of OCT4-positive cells. Different letters in the.

last column indicate significant differences (***p*** < 0.05). S: somites.+: positive cells. −: Not detected. ND: not done.

**Figure 3:**
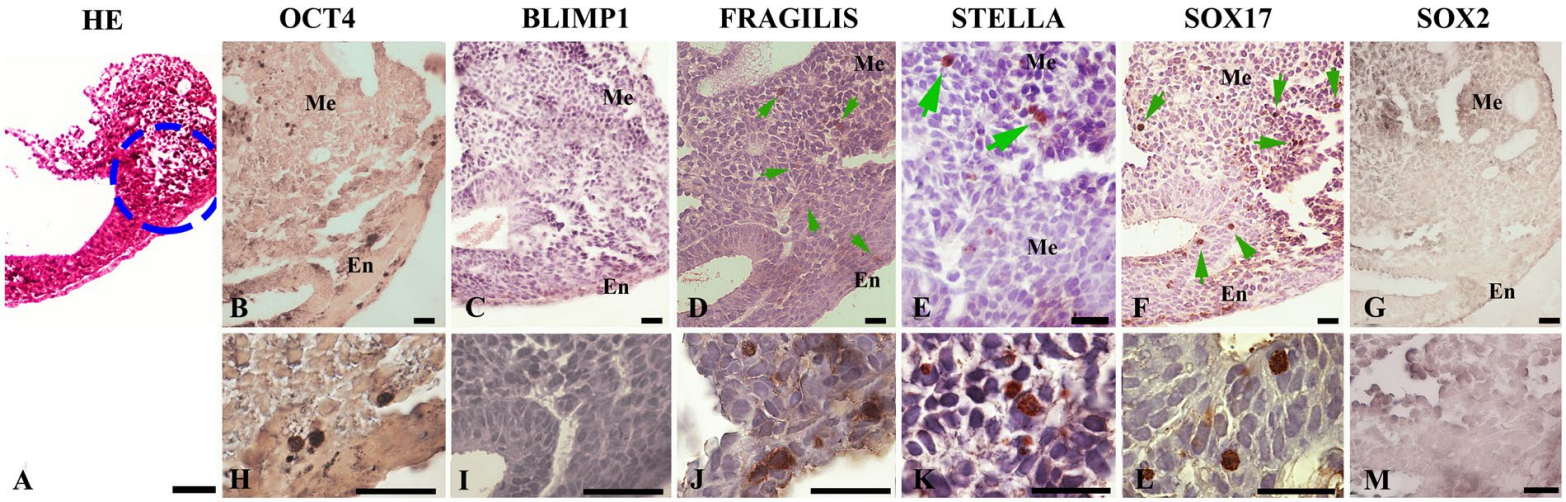
Germ line marker expression in the neural plate stage of the *Lagostomus maximus* embryo. (**A**) Hematoxylin-eosin (HE) stained general view of the neural plate stage; blue dotted-circle indicates immunoreactive areas in which markers were found. (**B**) OCT4-, (**D**) FRAGILIS-, (**E**) STELLA- and (**F**) SOX17-expressing cells in the base of the allantois in the ectoderm and mesoderm after gastrulation in the neural plate stage. (**C**,**I**) BLIMP1 and (**G**,**M**) SOX2 were not detected. (**H**–**M**) Details of the mesoderm area of the upper plate for each marker showing a higher magnification of presumptive PGC precursor cells positive for (**H**) OCT4, (**J**) FRAGILIS, (**K**) STELLA and (**L**) SOX17 and negative for (**I**) BLIMP1 and (**M**) SOX2. Me: mesoderm; En: endoderm. Scale bar: 20 µm (**B–D**,**F**,**G**); 40 µm (**A**,**E**,**M**); 1,000 µm (**H**–**L**).

In the neural plate stage, with the emergence of the allantoic bud, *OCT4*, *SOX17*, *FRAGILIS* and *STELLA* expression was detected in a subset of cells of the posterior epiblast (Fig. 3B,D–F,H,J–L). Importantly, these cells were located in the posterior streak in the mesoderm and endoderm of the wall of the visceral endoderm at an angle with the allantois. Interestingly, during these satages, BLIMP1 and SOX2 proteins were undetectable (Fig. 3C,G,I,M).

**Figure 4:**
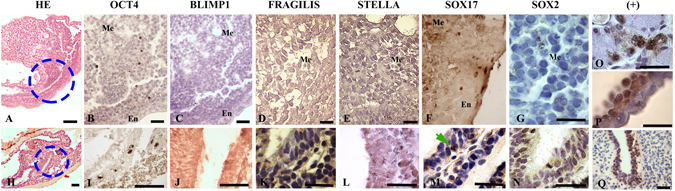
Localization of germ line marker-expressing cells in the early- and late-head fold stages in *Lagostomus maximus* embryos. Hematoxylin-eosin stained (HE) general views of (**A**) early- and (**H**) late-head fold stages showing areas in which the indicated markers were detected in the upper and lower plates, respectively (blue dotted circles). (**B**) OCT4-positive, (**C**) BLIMP1-negative, (**D**) FRAGILIS-negative, (**E**) STELLA-negative, (**F**) SOX17-positive and (**G**) SOX2-negative cells in the early-head fold embryo. (**I**) OCT4-positive, (**J**) BLIMP-negative, (**K**) FRAGILIS-negative, (**L**) STELLA-negative, (**M**) SOX17-positive (green arrows) and (**N**) SOX2-negative migratory PGCs in the endoderm in the late-head fold pre-somite embryo. Positive control (+): (**O**) BLIMP1 expression in tonsil tissue, (**P**) SOX17 expression in the visceral endoderm and (**Q**) SOX2 expression in neural tube tissue, all from *L. maximus*. Me: mesoderm; En: endoderm. Scale bar: 20 µm (**H**); 40 µm (**A**–**F**,**I**,**Q**); 1,000 µm (**G**,**J**–**N**,**O**,**P**).

In early head-fold, *OCT4-* and *SOX17*-positive cells were observed as in the previous stage (Fig. 4B,F). However, at the late head-fold stage, during translocation to the hindgut, *OCT4* expression was positive, and *SOX17* expression was negative except for in a few cells (Fig. 4I,M). The expression of *BLIMP1*, *STELLA*, *SOX2* and *FRAGILIS* at this stage was undetectable (Fig. 4C–E,G,J–L and N). As BLIMP1 and SOX2 proteins were not detected at any time-point, positive controls were performed using tissues in which BLIMP1 (Fig. 4O), SOX2 (Fig. 4P) and SOX17 (Fig. 4Q) were known to be expressed, showing that the antibodies used were able to recognize proteins from *L. maximus* tissues.

**Figure 5:**
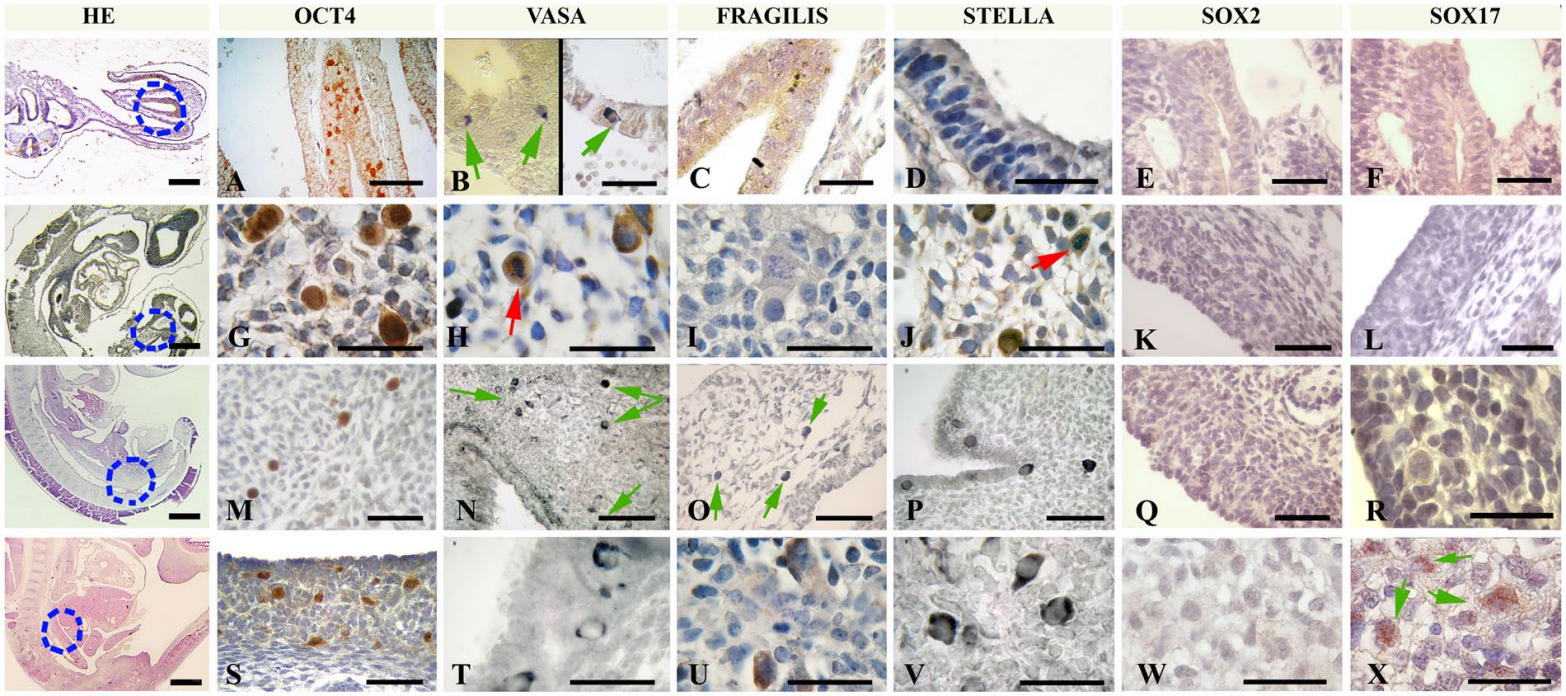
Detection of germ line markers in cells migrating through the dorsal mesentery and colonizing the genital ridge. (**A**) OCT4- and (**B**) VASA-expressing migratory-PGCs in the developing hindgut endoderm in embryos with 8–12 pairs of somites; (**C**) FRAGILIS, (**D**) STELLA, (**E**) SOX2 and (**F**) SOX17 were not detectable at this stage. (**G**) OCT4-, (**H**) VASA- and (**J**) STELLA-expressing migratory-PGCs in the mesentery in embryos with 25–30 pairs of somites; red arrows indicate mitotic dividing migratory-PGCs (**I**) FRAGILIS, (**K**) SOX2 and (**L**) SOX17 were not detected at this stage. (**M**) OCT4, (N) VASA, (**O**) FRAGILIS and (**P**) STELLA positive migratory-PGCs in the mesentery in embryos with 35–40 pairs of somites; (**Q**) SOX2 and (**R**) SOX17 were not detected at this stage. (**S**) OCT4-, (**T**) VASA-, (**U**) FRAGILIS-, (**V**) STELLA- and (**X**) SOX17-positive migratory-PGCs colonizing the genital ridge in embryos with >50 pairs of somites; (**W**) SOX2 was not detected at this stage. HE column: general view of embryo sagittal sections showing the area detailed in the corresponding lines (blue dotted circles). Green arrows indicate immune-positive cells. Scale bar: 20 µm (HE column); 40 µm (**A**–**C**,**E**,**F**,**K**–**Q**,**S**); 1,000 µm (**D**,**G**–**J**,**R**,**T**–**X**).

**Figure 6:**
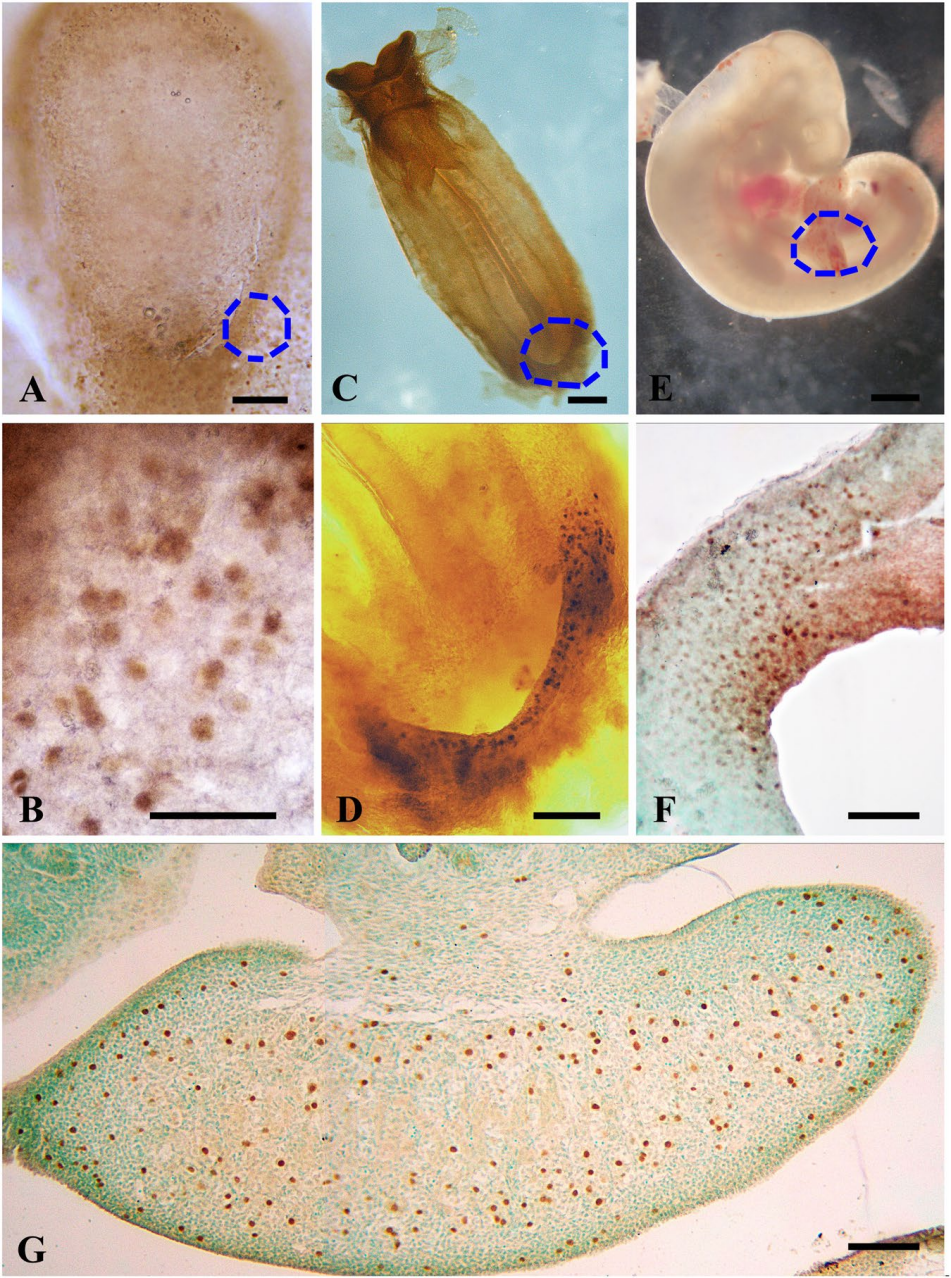
Whole-mount embryo immunohistochemistry: detection of germ line markers during PGC migration and colonization in the *L. maximus* embryo. (**A**) Embryonic disc showing OCT4-positive cells at the pre-somite stage, (**B**) details of the blue dotted circle in (**A**) showing OCT4-positive cells in the epiblast. (**C**) An embryo with 8 pairs of somites showing OCT4-positive cells at the base of the allantois; (**D**) detail of the blue dotted area in (**B**). (**E**) General view of an embryo with >20 pairs of somites, (**F**) details of OCT4-expressing migratory-PGCs in the hindgut (blue dotted circle in **E**). (**G**) Sagittal section of the fetal ovary in an embryo with >50 pairs of somites showing colonization of OCT4-positive PGCs (brown staining; counterstaining with methyl green); note that PGCs are still arriving to the ovary.

During early migration, *OCT4*-expressing cells were found in the gut-mesentery in embryos with 8–12 pairs of somites (Figs 5A and 6B,E). Unexpectedly, the expression of *VASA* was positive in the cytoplasm of translocated cells at this early stage (Fig. 5B), and SOX2, SOX17, STELLA and FRAGILIS were undetectable at this stage (Fig. 5C–F). OCT4-positive cells were observed in the dorsal mesentery through the hindgut in embryos with 35 pairs of somites, in embryos with more than 45 pairs of somites (Figs 5G,M and 6F) and during the colonization of the genital ridges in embryos with more than 60 somites (Figs 5S and 6G). Cells positive for *VASA* and *STELLA* expression were seen during migration in embryos with more than 30 pairs of somites in the dorsal mesentery through the hindgut and during the colonization of the genital ridges (Fig. 5H,I,N,O,T and U). *FRAGILIS* expression was detected late during migration in embryos with more than 45 somites (Fig. 5P) and during the colonization of the genital ridges (Fig. 5V).

*BLIMP1* (data not shown) and *SOX2* expression was undetectable during migration and colonization of the genital ridges (Fig. 5E,K,Q and W). Interestingly, *SOX17* expression was not detected during migration (Fig. 5F,L and R), but when the genital ridges were colonized, a low SOX17-positive signal was observed (Fig. 5X).

#### Germ cell quantification during migration and genital ridge colonization

The number of *OCT4*-expressing cells was estimated from the embryo disc until fetal ovary colonization. Estimates based on OCT4-expressing cells are shown in Table 1; other detectable markers for each developmental stage are also indicated.

At the embryo disc and primitive streak stages, OCT4-positive cells were abundant, but no other marker was detected at this developmental time-point (Fig. 6A,D). At the neural plate stage, a small group of 8–12 OCT4-expressing cells was identified in the mesoderm. From the late head-fold stage, approximately 25 cells retaining *OCT4* expression started to migrate increasing rapidly in number to a hundred cells located at the gut-mesentery (Fig. 6B,E). More than 1,000 cells were detected by the end of migration rapidly increasing to a total of approximately 55,000 cells once fetal ovary colonization was achieved (Fig. 6G).

## Discussion

### The basal rodent *L. maximus* develops through the mammotypical embryo disc

In this study, we show that the basal Hystricognathi rodent *L. maximus* develops through a flat disc, where no contact between the ExE and the epiblast exists. The topological organization of the pre-gastrulating embryo determines a scenario for PGC formation divergent from the mouse model. At the beginning of implantation, the pre-gastrulating *L. maximus* embryo shows an ICM ball positioned at the distal part of the conceptus, which then begins to transform into a flat-disc epiblast, far apart from the trophoblast located at the proximal part of the conceptus. This topology departs from that found in the rodent embryo model based on laboratory mice, in which the epiblast makes close contact with the ExE and both tissues are enveloped by the VEn, defining the typical mouse egg-cylinder morphology^16^. In *L. maximus* the epiblast and the amniotic cavity develop from a bilaminar disc by cavitation rather than by folding. The flat disc epiblast morphology of the *L. maximus* embryo is shared with non-rodent mammals that have been studied so far, such as rabbits, pigs, cows, and humans among others^12^, and it is considered the mammotypical embryo morphology. It is worth noting that *L. maximus* epiblast morphology resembles that of human embryos, where the epiblast does not contact the trophoblast and remains separated from it by the pro-amniotic cavity before and during gastrulation, whereas in the majority of non-rodent mammals the epiblast fuses laterally with the mural trophoblast as a consequence of the disappearance of the polar layer of trophoblast cells^12^.

### Cells expressing mammalian PGC-associated proteins originate from mesoderm progenitors in *L. maximus*

OCT4 protein seems to play an essential role in the establishment and maintenance of the germ line. *OCT4* expression in the pre-gastrulating embryo was uniform in the epiblast cells, but after the primitive streak stage, *OCT4* was mostly down-regulated, and its expression only persisted in a group of cells that was later restricted to the mesoderm of the posterior end of the embryo. It seems likely that *OCT4* expression is required for maintaining pluripotency, helping to epigenetically reprogram cells for PGC development that will be specified at a later stage (see below). Pluripotent genes, such as *Nanog*, *Sox2* and *Oct4*, are restored at E7 to E7.75 in the mouse embryo during early gastrulation, suppressing expression of genes involved in mesodermal specification^17–19^.

In mice, *Blimp1* orchestrates somatic gene repression and promotes pluripotency genes^20, 21^. Despite using two different antibodies and validating them through protein recognition in *BLIMP1*-expressing tissues of *L. maximus*, we could not detect its expression during early gastrulation or later stages of development. Because failure to detect the protein is not evidence of its absence, especially as we did not carry out experiments to define the threshold of protein detection under our conditions of immunohistochemistry, further analysis is still needed to assess when and where *BLIMP1* is expressed in *L. maximus* or if it is even expressed at all. The essential role played by *Blimp1* in mouse germ cell specification has also been reported in rabbit embryos^22^, but its role has been considered unlikely in other flat embryos, such as those of pigs^23^. In humans, *SOX17* rather than *BLIMP1* seems to be the key regulator of PGC destination^24^, although recent *in vitro* studies also suggest a role for *BLIMP1* in human PGC-like cells derived from induced pluripotent stem cells^25^. As in the human embryo, we did detect *SOX17* instead of *BLIMP1* in the flat disc of *L. maximus*; when mesodermal OCT4-positive cells become restricted in number, they begin to express *SOX17*. This advocates for a comparable situation in the regulation of PGC fate in *L. maximus* and human embryos. In early mouse PGC development, *SOX2* instead of *SOX17* protein seems to accomplish this essential role; similar to human PGC specification, we did not find *SOX2* expression in *L. maximus* ^24, 26^. It is likely that the temporal co-localization of SOX17 and OCT4 proteins in *L. maximus* PGCs plays a major role in inhibiting somatic genes and maintaining pluripotency because alternative SOX/OCT4 pairings target specific loci to regulate pluripotent cell fate and differentiation programs^27^. During migration, *SOX17* is down-regulated, and its expression is restored in oogonia after the colonization of the genital ridges. It seems reasonable to consider that *SOX17* could act as a regulator of proliferation and the cell cycle^28^, contributing to the continuous rise of healthy germ cells, which characterizes the ovaries of *L. maximus* throughout fetal life, in the presence of a minimal rate of apoptosis-driven, germ cell attrition^29^.

At the neural plate stage, we detected transient expression of *FRAGILIS*, *STELLA* and *SOX17* in the proximal epiblast. In mice, the expression of both proteins seems to be necessary for the foundation of PGCs^4^. *Fragilis* expresses around the most proximal epiblast cells, and its expression intensifies in the posterior extra-embryonic mesoderm. *Stella* begins to express specifically in *Fragilis*-expressing cells in the extra-embryonic mesoderm and continues to be expressed in migrating PGCs. In contrast, in *L. maximus*, transiently expressed *FRAGILIS, STELLA* and *SOX17* turned on again at a later stage (35–39 somites) during migration.

The essential germ line marker VASA was expressed early in *L. maximus* during the translocation of OCT4-positive cells to the hindgut (8–12 somites). Thereafter, VASA-expressing cells were detected throughout the migration toward the genital ridges (cf. Figs 5 and 6). The expression of *VASA* was not observed before translocation to the hindgut (data not shown). VASA seems to be a major determinant of the germ cell for this species due to the fact that it is expressed very early in germ line development and continues to be expressed both in fetal and adult ovaries^30, 31^. In mice, the expression of VASA protein becomes detectable in PGCs at the late migrating stage in the gut mesentery of 9.5–10.5 dpc embryos^32^. This is also the case in the human embryo, where VASA expression is seen in PGCs migrating near the genital ridges^33, 34^.

The spatiotemporal pattern of expression of germ line markers found in *L. maximus* differs in many aspects from that described in mice, as discussed above. Moreover, it is difficult to understand this expression pattern in light of the currently accepted model on the origin of PGCs as a lineage-restricted cluster of cells in the base of the allantois, specified early just before, or during, gastrulation. In contrast, our results better accommodate an alternative model proposed by Mikedis and Downs^14^, in which PGCs are specified later from a pluripotent progenitor population within the embryonic axis. Before and during gastrulation, the *L. maximus* embryo showed a population of cells expressing the pluripotent protein OCT4 in the posteriorly extending embryo axis. Early on, at the 8–12 somites stage, these OCT4-positive cells translocated to the hindgut, the universal germ line marker VASA was expressed (cf. Table 1 and Fig. 5), and then, STELLA and FRAGILIS proteins were identified in between the 25–30 somites and 35–39 somites stages (cf. Fig. 5J and Table 1). It seems reasonable to speculate that OCT4/STELLA/FRAGILIS-expressing cells within the migrating pluripotent population finally are restricted to form PGCs once the evolutionarily conserved germline-specific VASA protein is expressed^14^.

From a few 8–12 OCT4-positive cells at the neural plate stage, a cluster of approximately 25–30 cells was found at the beginning of migration in *L. maximus*, which is comparable to what has been described in mice and humans. As these cells proliferate during migration, they increase to approximately 1,200 when entering the genital ridges, a comparable number of colonizing PGCs to that seen in humans^35^.

### Evolutionary considerations

The mouse egg-cylinder morphology has been assumed as the typical early post-implantation rodent embryo; however, both *L. maximus*, as well as *Cavia porcellus*, depart from this generalized morphology. Together with Sciuromorpha, Hystricognathi are the first offshoots in the rodent phylogeny^36, 37^ after the separation of Rodentia and Lagomorpha^38^, whereas Myomorpha, especially the mouse-related clade Muroidea, are the most recently evolved rodents^39^. The planar epiblast morphology in the basal *L. maximus* supports the idea that the egg-cylinder in which the ExE comes in close contact with the epiblast is an innovation of more recently evolved rodents such as murids rather than a characteristic of the entire order^20^. The appearance of the ExE, deriving from the persistent polar trophoblast, and the germ layer inversion that is provoked as the embryo sinks into the yolk-cavity are the landmarks of the early implantation rodent embryo compared with those of other mammals. This phenomenon occurs in different ways in the few species belonging to the different suborders of Rodentia that have been analyzed so far, and the ExE can be recognized in all cases^40^. The extensive embryo invagination into the yolk-cavity seen in mice, which enables close contact of the ExE and epiblast, does not occur in *L. maximus*, as shown in this study, or in its close evolutionary relative *C. porcellus* ^13^. Moreover, this seems to be the case in other basal rodents, such as the Sciuromorpha *Spermophilus tridecemlineatus*, and even in species from basal offshoots of the Myomorpha clade, such as *Geomys bursarius* ^40^.

Our observation that PGCs are specified late in *L. maximus* is supported by the recent proposal by Johnson and Alberio^23^ that the precocious (before gastrulation) specification of the germ line seen in mice enables an accelerated development of somatic innovations favoring speciation and the characters of typical r-strategists, *i.e*., short gestations, large litters, small body-size, and a short life-span. The key for early germ line restriction in mice resides in the appearance of the ExE and its capacity to produce germ line inducing proteins^23^. This may well apply to the Muroidea clade but not to other clades in Rodentia, such as Hystricognathi. In *L. maximus*, as well as in *C. porcellus*, the ExE remains far apart from the epiblast before and after gastrulation, indicating that it would not be involved in germ line inducing signaling.

Finally, it is worth highlighting that the pattern of expression of PGC-associated markers in *L. maximus* described in this report diverges from that in mice and might be relevant to other non-rodent mammals with planar embryo morphology, including humans. The early implantation embryo of *L*. *maximus* resembles the human embryo in that *SOX17* rather than *BLIMP1* seems to play a central role in the fate of PGCs^21^, SOX2 is not involved in PGC specification^24^ and both embryos have a comparable planar morphology differing from other embryo-disc developing mammals^12^.

## Methods

### Animals

Experimental protocols concerning animals were approved by the Institutional Committee on the Use and Care of Experimental Animals (CICUAE-Universidad Maimónides). The handling and killing of animals was performed in accordance with the standards defined by the Guide for the Care and Use of Laboratory Animals (CCAC 2002) and Guidelines on the Care and Use of Wildlife (CCAC 2003) from the Canadian Council of Animal Care. A total of 99 pregnant adult female plains vizcacha, *Lagostomus maximus*, (>2.5 kg) were captured over three consecutive years during the main breeding season (from March-April to August) using live traps located at the entrance of burrows from a natural resident population at the Estación de Cría de Animales Silvestres (ECAS), Ministry of Agriculture, Villa Elisa, Buenos Aires province, Argentina. The capture and transport of animals were approved by the Ministry of Agriculture Authority of the Buenos Aires Province Government.

### Sample collection

A total of 198 embryos/fetuses were recovered and analyzed. Only the embryo located nearest the cervix from each uterine horn was included in this analysis, due to the fact that they are the only ones that develop to term; the remaining anterior-implanted embryos stop developing at early post-implantation stages and are selectively aborted^41, 42^. Embryo development of *L. maximus* was analyzed from the beginning of implantation up to the >60 pairs of somites stage. Post implantation embryo development was rated per week and placed in a relative chronological sequence. Organization and classification of the embryonic stages were established on a comparative basis with mice and close evolutionarily related chinchillas and guinea pigs^43–46^ using Theiler Stages^43^. Developmental data were supplemented with the capture-time, length and width of the implantation site, gross embryo morphology, somite number, and crown-heel length, width and weight in more developed fetuses.

### Tissue collection and histological preparation

Pregnant females were anaesthetized by the intramuscular administration of 13.5 mg/kg body weight of ketamine chlorhydrate (Holliday Scott S.A., Buenos Aires, Argentina) or 0.6 mg/kg body weight xylazine chlorhydrate (Richmond Laboratories, Veterinary Division, Buenos Aires, Argentina) and euthanized using an intracardiac injection of Euthanyl (0.5 ml/kg body weight, sodium pentobarbital, sodium diphenylhydantoin, Brouwer S.A., Buenos Aires, Argentina). Uterine horns were exposed, removed and thoroughly rinsed in PBS, pH 7.4. The IS and the embryos/fetuses were removed from the horns, measured and fixed in cold 4% neutral-buffered *para*-formaldehyde (PFA) for 24 h. PFA-fixed tissues were dehydrated through a graded series of ethanol (50%, 70%, 95% and 100%), embedded in paraffin, serially sectioned at 6 μm, and mounted onto cleaned coated slides. Sections were dewaxed in xylene (Sigma Chemical Co., St. Louis, MO, USA) and re-hydrated through a series of decreasing concentrations of ethanol. At least 3 to 5 slides of each specimen were stained with haematoxilyn-eosin for general histology inspection. The remaining consecutive serial-sectioned slides were stored at room temperature until used for immunohistochemistry.

### Immunohistochemistry

Dewaxed and re-hydrated sections were subjected to blocking of endogenous peroxidase activity with 3% H_2_O_2_ for 20 min at room temperature. They were placed in sodium citrate buffer (10 mM sodium citrate, 0.05% Tween-20, pH 6.0) for heat-induced epitope retrieval for 20 min in a water bath set at 100 °C. The sections were incubated with a blocking solution containing 10% bovine fetal serum in PBS (pH 7.4) for 30 min at room temperature. Immunoreactivity was detected by incubating the slides overnight at 4 °C with specific rabbit polyclonal anti-FRAGILIS IgG (1:250, ab15592, Abcam, Cambridge, UK), anti-OCT4 IgG (1:250, ab19857, Abcam, Cambridge, UK), anti-DDX4/MVH IgG (1:250, ab13840, Abcam, Cambridge, UK), anti-STELLA IgG (1:250, ab19878, Abcam, Cambridge, UK) or anti-SOX17 IgG (1:250, ab155402, Abcam, Cambridge, UK). To analyze the expression of BLIMP1, two different primary antibodies were used: polyclonal goat anti-BLIMP1 (H-150) IgG (1:100, sc-25380, Santa Cruz Biotechnology) and a purified polyclonal rat anti-Human/Mouse BLIMP1 (1–50, 14–5923 Affymetrics, eBiosciences, Santa Clara, CA, USA). Goat polyclonal anti-SOX2 (Y-17) antibody IgG (1:100, sc-17320, Santa Cruz Biotechnology, Santa Cruz, CA, USA) was kindly provided by Dr. Ramiro Alberio (University of Nottingham, UK). The immune reaction was revealed with the appropriate biotinylated secondary antibodies, *i.e*., anti-rabbit IgG, anti-goat IgG or anti-rat IgG, followed by incubation with an avidin–biotin complex (ABC Vectastain Elite Kit, Vector Laboratories, Burlingame, CA, USA). The reaction was visualized with DAB (SK-4100, DAB Kit, Vector Laboratories, Burlingame, CA, USA) or with DAB blue (SK-4700, DAB Kit, Vector Laboratories, Burlingame, CA, USA). Finally, the treated sections were dehydrated through a graded series of ethanol (70, 95 and 100%), cleared in Neo-Clear (Merck, Darmstadt, Germany) and covered using coverslips. Staining for each antibody was repeated at least three times in separate assays for each specimen, using a minimum of two slides per assay. Repetitions of the assays, performed on different days, confirmed that staining was reproducible. For each embryo stage, all antibodies were screened in serial sections on the same slide. Positive controls for SOX2, SOX17, and BLIMP1 included neural tube, fetal lung or visceral endoderm, and tonsil tissue from *L. maximus*, respectively, and were simultaneously processed with the primary antibodies. Negative control assays were carried out by pre-absorbing primary antibodies with synthetic peptides (FRAGILIS ab15737, OCT4 ab20650, DDX4/MVH ab13841 and STELLA ab23324, Abcam, Cambridge, UK) or by omitting the primary antibody (BLIMP1, SOX17, SOX2).

### Embryo whole mount immunohistochemistry

Whole embryos were PFA-fixed for 4 h at 4 °C and then washed three times in PBT (0.2% Tween-20 in PBS) for 15 min at 4 °C. Embryos were subjected to permeabilization through a series of methanol (25%, 50%, 75%, 90% and 100%) in PBT for 10 min at 4 °C. Embryos were kept at −20 °C overnight and then were subjected to rehydration through 10 min washes in a decreasing series of methanol (90%, 75%, 50% and 25%) in PBT at 4 °C, followed by a final 10 min wash in PBT. Embryos were blocked for endogenous peroxidase activity with 5% H_2_O_2_ in methanol for 5 h at room temperature and then incubated for 1 h in PBS-MT blocking solution (PBT + 2% w/v nonfat dry milk) at room temperature on a shaker. Immunoreactivity was achieved by incubating the embryos overnight at 4 °C with a specific anti-OCT4 IgG (1:250, ab19857, Abcam, Cambridge, UK) diluted in PBS-MT on a shaker and washed 5 times for 1 h in PBS-MT. The immune reaction was revealed with biotinylated anti-rabbit IgG overnight with rotation at 4 °C, washed five times for 1 h in PBS-MT, incubated with an avidin–biotin complex (ABC Vectastain Elite Kit, Vector Laboratories, Burlingame, CA, USA) with rotation for 30 min and washed five times for 1 h in PBS-MT. The reaction was visualized with DAB (SK-4100, DAB Kit, Vector Laboratories, Burlingame, CA, USA). Finally, the treated sections were washed three times in PBS-MT once the reaction and staining had reached the desired intensity. Samples were observed and photographed on excavated slides in PBT. They were then preserved in glycine until used for sectioning.

### Germ cell quantification and statistical analysis

The total numbers of OCT4-positive cells during gastrulation, migration and genital ridge colonization were quantified. Each embryo was completely cut into 6 μm-serial sections. All the embryo sections were counted for each embryo. A cell count was performed in 3 whole embryos for each developmental stage from neural plate to <30 somites. Embryos with >30 somites were quantified using a stereoscopic method, as previously reported^26^. Cell counting was expressed as the mean ± S.D. GraphPad Prism software (version 5.0 for Windows GraphPad Software, San Diego, CA, USA) was used for a one-way analysis of variance. A Newman-Keuls test was used when differences between more than two groups were compared. A *p*-value of less than 0.05 was considered statistically significant.

## Electronic supplementary material

Supplementary Information

## References

[1] Bertocchini F, Chuva de Sousa Lopes SM (2016). Germline development in amniotes: A paradigm shift in primordial germ cell specification. Bioessays.

[2] Molyneux K, Wylie C (2004). Primordial Germ Cell Migration. Int. J. Dev. Biol..

[3] Saitou, M. & Yamaji, M. Primordial germ cells in mice. *Cold Spring Harb Perspect Biol*. **4**, pii:a008375 (2012). 10.1101/cshperspect.a008375 PMC3536339 23125014

[4] Saitou M, Barton SC, Surani MA (2002). A molecular programme for the specification of germ cell fate in mice. Nature.

[5] Surani MA, Hayashi K, Hajkova P (2007). Genetic and epigenetic regulators of pluripotency. Cell.

[6] Tres LL, Rosselot C, Kierszenbaum AL (2004). Primordial germ cells: what does it take to be alive?. Mol Reprod Dev..

[7] Lawson KA (1999). Bmp4 is required for the generation of primordial germ cells in the mouse embryo. Genes Dev..

[8] Ohinata Y (2005). *Blimp1* is a critical determinant of the germ cell lineage in mice. Nature.

[9] Ginsburg M, Snow M, McLaren A (1990). Primordial germ cells in the mouse embryo during gastrulation. Development.

[10] Lawson KA, Hage WJ (1994). Clonal analysis of the origin of primordial germ cells in the mouse. Ciba Found. Symp..

[11] Bowles J, Koopman P (2007). Retinoic acid, meiosis and germ cell fate in mammals. Development.

[12] Sheng G (2015). Epiblast morphogenesis before gastrulation. Dev. Biol..

[13] Chuva de Sousa Lopes SM, Roelen BA (2008). Primordial germ cell specification: the importance of being “blimped”. Histol. Histopathol..

[14] Mikedis MM, Downs KM (2014). Mouse primordial germ cells: A reappraisal. Int. Rev. Cell Molec. Biol..

[15] Downs KM, Inman KE, Jin DX, Enders AC (2009). The Allantoic Core Domain: new insights into the development of the murine allantois and its relation to the primitive streak. Dev. Dyn..

[16] Bedzhov I, Zernicka-Goetz M (2014). Self-organizing properties of mouse pluripotent cells initiate morphogenesis upon implantation. Cell.

[17] Campolo F (2013). Essential role of Sox2 for the establishment and maintenance of the germ cell line. Stem Cells.

[18] Chambers I (2007). Nanog safeguards pluripotency and mediates germline development. Nature.

[19] Kehler J (2004). Oct4 is required for primordial germ cell survival. EMBO Rep..

[20] Kurimoto K (2008). Complex genome-wide transcription dynamics orchestrated by Blimp1 for the specification of the germ cell lineage in mice. Genes Dev..

[21] Robert VJ, Garvis S, Palladino F (2015). Repression of somatic cell fate in the germline. Cell. Mol. Life Sci..

[22] Hopf C, Viebahn C, Püschel B (2011). BMP signals and the transcriptional repressor *BLIMP1* during germline segregation in the mammalian embryo. Dev. Genes Evol..

[23] Johnson AD, Alberio R (2015). Primordial germ cells: the first cell lineage or the last cells standing?. Development.

[24] Irie N (2015). SOX17 is a critical specifier of human primordial germ cell fate. Cell.

[25] Sasaki K (2015). Robust *In Vitro* Induction of Human Germ Cell Fate from Pluripotent Stem Cells. Cell Stem Cell.

[26] De Jong J (2008). Differential expression of SOX17 and SOX2 in germ cells and stem cells has biological and clinical implications. J. Pathol..

[27] Aksoy I (2013). Sox transcription factors require selective interactions with Oct4 and specific transactivation functions to mediate reprogramming. Stem Cells.

[28] Ye YW (2011). Sox17 regulates proliferation and cell cycle during gastric cancer progression. Cancer Lett..

[29] Inserra PIF, Leopardo NP, Willis MA, Freysselinard A, Vitullo AD (2014). Quantification of healthy and atretic germ cells and follicles in the developing and post-natal ovary of the South American plains vizcacha, *Lagostomus maximus*: evidence of continuous rise of the germinal reserve. Reproduction.

[30] Jensen FC, Willis MA, Albamonte MS, Espinosa MB, Vitullo AD (2006). Naturally suppressed apoptosis prevents follicular atresia and oocyte reserve decline in the adult ovary of *Lagostomus maximus* (Rodentia, Caviomorpha). Reproduction.

[31] Leopardo NP, Jensen F, Willis MA, Espinosa MB, Vitullo AD (2011). The developing ovary of the South American plains vizcacha, *Lagostomus maximus* (Mammalia, Rodentia): massive proliferation with no sign of apoptosis-mediated germ cell attrition. Reproduction.

[32] Noce T, Okamoto-Ito S, Tsunekawa N (2001). Vasa homolog genes in mammalian germ cell development. Cell Struct Funct.

[33] Raz, E. The function and regulation of vasa-like genes in germ-cell development. *Genome Biol*. **1**, doi:10.1186/gb-2000-1-3-reviews1017, reviews1017 (2000). 10.1186/gb-2000-1-3-reviews1017 PMC138859 11178242

[34] Castrillon DH, Quade BJ, Wang TY, Quigley C, Crum CP (2000). The human VASA gene is specifically expressed in the germ cell lineage. Proc. Natl. Acad. Sci..

[35] De Felici, M. Origin, migration, and proliferation of human primordial germ cells. In *Oogenesis* (ed. G. Coticchio, D.F. Albertini & L. De Santis) 19–37 (London: Springer-Verlag, 2013).

[36] Montgelard C, Forty E, Arnal V, Matthee CA (2008). Suprafamilial relationships among Rodentia and the phylogenetic effect of removing fast-evolving nucleotides in mitochondrial, exon and intron fragments. BMC Evol Biol..

[37] Churakov G (2010). Rodent Evolution: Back to the Root. Mol. Biol. Evol..

[38] Voloch CM, Vilela JF, Loss-Oliveira L, Schrago CG (2013). Phylogeny and chronology of the major lineages of New World hystricognath rodents: insights on the biogeography of the Eocene/Oligocene arrival of mammals in South America. BMC Research Notes.

[39] Fabre PH, Hautier L, Dimitrov D, Douzery EJP (2012). A glimpse on the pattern of rodent diversification: a phylogenetic approach. BMC Evol. Biol..

[40] Ozdzenski W, Mystkowska ET (1976). Implantation and early postimplantation development of the bank vole *Clethrionomys glareolus*, Schreber. J. Embryol. Exp. Morphol..

[41] Roberts CM, Weir BJ (1973). Implantation in the plains vizcacha, *Lagostomus maximus*. J Reprod Fert.

[42] Jensen FC, Willis MA, Leopardo NP, Espinosa MB, Vitullo AD (2008). The ovary of the gestating South American plain vizcacha (*Lagostomus maximus*): supressed apoptosis and corpora lutea persistence. Biol. Reprod..

[43] Theiler, K. *The House Mouse - Atlas of Embryonic Development* (Springer-Verlag, 1989).

[44] Kaufman, M. H. Morphological stages of postimplantation embryonic development in *Postimplantation Mammalian Embryos: A Practical Approach* (ed. A.J. Copp & D. L. Cockroft) 81–89 (Oxford: IRL Press at Oxford University Press, 1990).

[45] Tibbitts FD, Hillemann HH (1959). The development and histology of the chinchilla placentae. J morphol..

[46] Wimsatt WA (1975). Some comparative aspects of implantation. Biol Reprod.

