# Supplementary material for: Early embryonic development and spatiotemporal localization of mammalian primordial germ cell-associated proteins in the basal rodent Lagostomus maximus
Source: Sci Rep. 2017 Apr 4;7:594. doi: 10.1038/s41598-017-00723-6 (PMC5429608; doi:10.1038/s41598-017-00723-6)
Supplement: Supplementary file 1 — Supplementary Information [file 41598_2017_723_MOESM1_ESM.pdf]

**SUPPLEMENTARY INFORMATION**

# Early embryonic development and spatiotemporal localization of mammalian primordial germ cell-associated proteins in the basal rodent *Lagostomus maximus*

Noelia P. Leopardo and Alfredo D. Vitullo

Centro de Estudios Biomédicos, Biotecnológicos, Ambientales y Diagnóstico, CEBBAD, Universidad Maimónides, Buenos Aires, Argentina

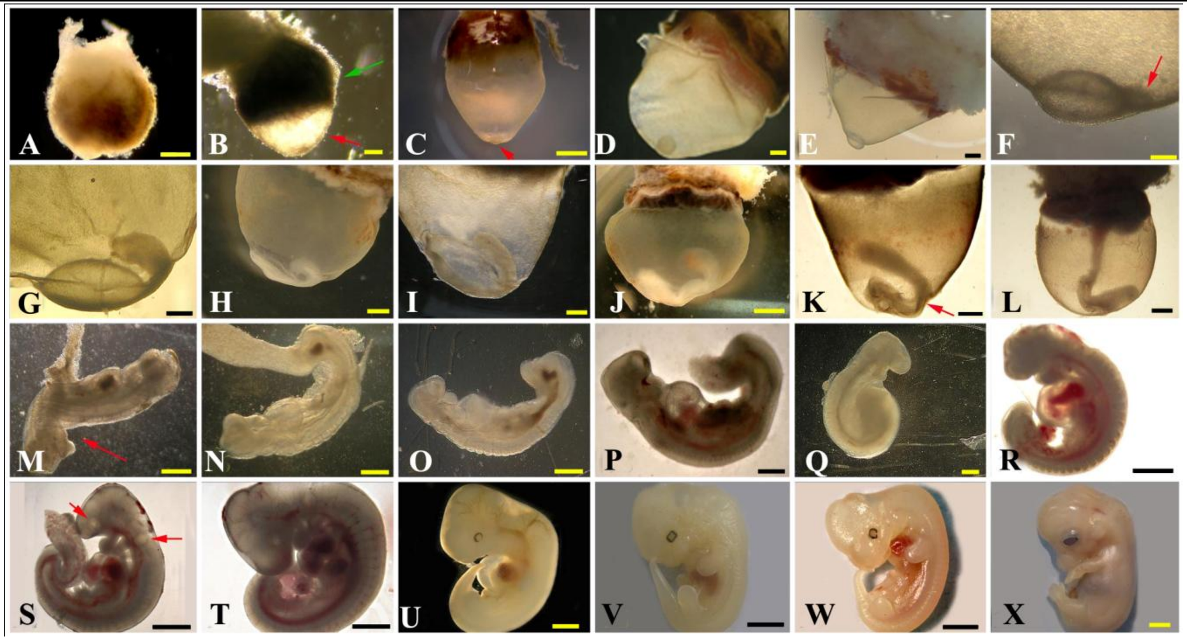


**Supplementary Figure 1.** General morphogenesis on fresh post-implantation conceptuses in *Lagostomus maximus*. (A) Newly implanted embryos (22-26 days) are round-shaped, (B) soon differentiating a pro-amniotic cavity (red arrow) and a throphoblast (green arrow). (C, D) At pre-streak stage, the embryo acquires a cup-shaped aspect with visible inner cell mass (red arrow). (E) At the beginning of gastrulation, first evidence of embryonic axis appears and amniotic cavity develops, (F) soon followed by allantoid bud appearance (red arrow) at neural plate stage. (G) At early head-fold pre-somite stage, the allantoids bud enlarges and (H, I) the cephalic region begins to turn. (J-L) With the appearance of the first somites, the allantoid extends through the exocoelomic cavity until it contacts the chorion; note the foregut pocket (red arrow in K). (M) Embryo turning initiates (red arrow) and (N-R) turned embryo progressively increases in somite number; (S) at 30-40 pairs of somites, optic and otic vesicles are evident (red arrows), (T) umbilical hernia is visible and (U) the tail elongates. (V-X) From 45 to 60 pairs of somites lens vesicles close, the eye is defined and individual fingers are visible. Scale bar: 100 µm (A, B); 250 µm (C-E, G); 500 µm (F, H, I, M-P), 1,000 µm (J- L, Q-T); 2,000 µm (U-X).

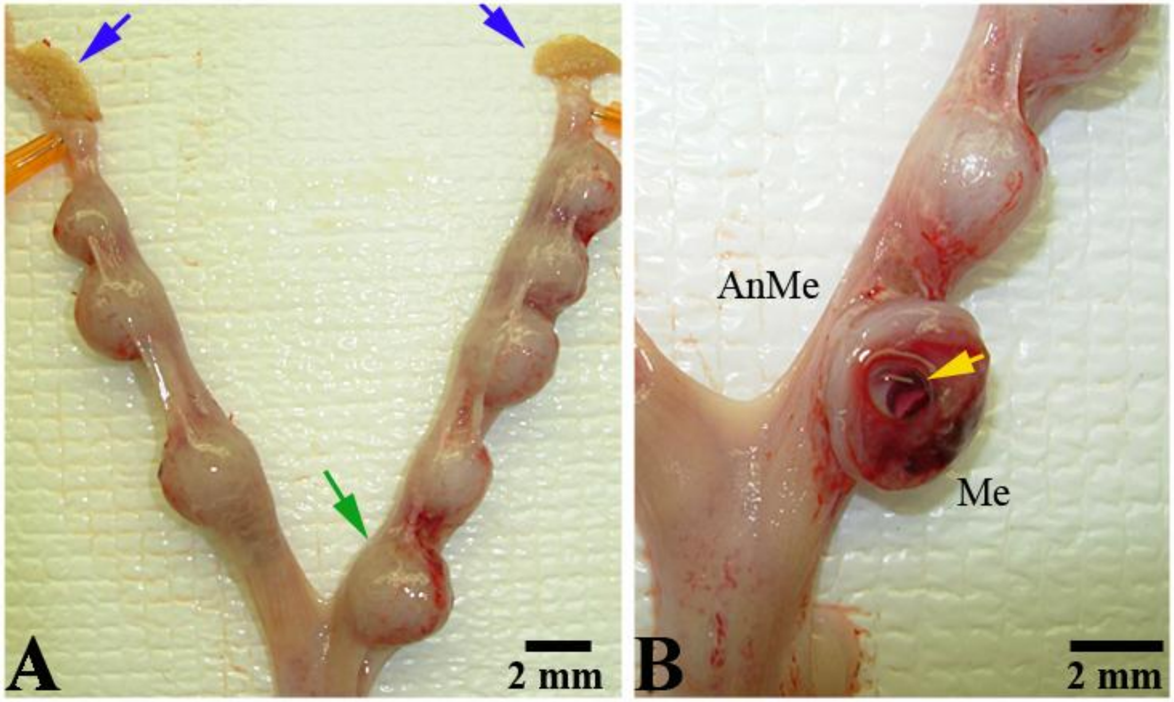


**Supplementary Figure 2**. Implantation site in the bicornuate uterus of *Lagostomus maximus*. (A) Uterine horns with 8 implantation sites (green arrow) at an early stage of pregnancy (22-26 days post-fertilization); ovaries are indicated by blue arrows. (B) Cutaway view of the uterus showing the blastocyst (yellow arrow) implanted with inner cell mass towards the mesometrial region.

**Supplementary Table 1**. Characterization and morphometrics of development in *L. maximus* embryo. Post-fertilization days were calculated based on an 18-day pre-implantation development according to Roberts & Weir (1973). Different letters in the same column indicate statistical differences ($p<0.05$).

| **Post fertilization** (day) | **Post implantation** (week) | **Number embryos/ fetus** | **Pairs of somites** | **Implantation Site (mm)** (mean ± S.D) | **Cephalocaudal length (mm)** (mean ± S.D) | **Weight (g)** (mean ± S.D) |
|---|---|---|---|---|---|---|
| 25 | 1 | 9 | - | 4,0 ± 0.02 [a] | - | - |
| | | 7 | - | 6,9 ± 0.02 [b] | - | - |
| 32 | 2 | 7 | - | 9,5 ± 0.72 [c] | - | - |
| | | 8 | - | 9,6 ± 0.71[c,d] | - | - |
| 39 | 3 | 6 | - | 11,1 ± 0.40 [e] | 2.5 ± 0.2[a] | - |
| | | 9 | 1-7 | 14,3 ± 0.84 [f] | 3.0 ± 0.1[a,b] | - |
| 46 | 4 | 8 | 8-12 | 14,4 ± 0.40[f,g] | 3.4 ± 0.0[a,b,c] | - |
| | | 9 | 13-20 | 16,2 ± 0.66 [h] | 3.6 ± 0.2[a,b,c,d] | - |
| 53 | 5 | 7 | 21-29 | 16,5 ± 0.65[h,i] | 4.5 ± 0.3[e] | - |
| | | 7 | 30-34 | 16,6 ± 0.49[h,i,j] | 5.8 ± 0.3[e,f] | - |
| 60 | 6 | 8 | 35-39 | 19,7 ± 0.47 [k] | 8.4 ± 0.4[g] | - |
| | | 8 | 40-44 | 20,3 ± 0.16 [k, l] | 9.5 ± 0.4[g,h] | - |
| 67 | 7 | 8 | >45 | 21,6 ± 0.43 [m] | 10.3 ± 0.4[i] | 0.3 ± 0.1[a] |
| | | | - | - | 13.7 ± 0.5[i,j] | 0.7 ± 0.1[a,b] |
| 74 | 8 | 9 | - | - | 19.1 ± 1.6[k] | 0.9 ± 0.1[a,b,c] |
| 81 | 9 | 9 | - | - | 29.7 ± 1.4[l] | 2.8 ± 0.4[d] |
| 88 | 10 | 8 | - | - | 45.3 ± 1.5[m] | 7.7 ± 0.6[e] |
| 95 | 11 | 7 | - | - | 51.7 ± 1.8[n] | 10.9 ± 1.0[e,f] |
| 102 | 12 | 6 | - | - | 57.9 ± 2.0[o] | 14.9 ± 1.5[g] |
| 107 | 13 | 9 | - | - | 68.9 ± 3.2[p] | 24.2 ± 1.9[h] |
| 112 | 14 | 8 | - | - | 86.3 ± 3.0[q] | 49.0 ± 4.8[i] |
| 119 | 15 | 8 | - | - | 103.8 ± 2.1[r] | 81.9 ± 5.8[j] |
| 126 | 16 | 7 | - | - | 112.8 ± 2.2[s] | 95.8 ± 5.6[k] |
| 133 | 17 | 9 | - | - | 122.4 ± 2.3[t] | 114.3 ± 6.1[l] |
| 140 | 18 | 9 | - | - | 134.6 ± 2.2[u] | 165.8 ± 5.3[m] |
| 147 | 19 | 8 | - | - | 153.1 ± 0.7[v] | 216.3 ± 4.47[n] |
